# The Vbeta13 T Cell Receptor Monoclonal Antibody Reduces Hyaluronan and CD68+, CD3+, and CD8+ Cell Infiltrations to Delay Diabetes in Congenic BB DR*Lyp/Lyp* Rats

**DOI:** 10.3389/fendo.2021.629242

**Published:** 2021-03-16

**Authors:** Marika Bogdani, Linda Faxius, Malin Fex, Anita Ramelius, Anya Wernersson, John P. Mordes, Elizabeth P. Blankenhorn, Åke Lernmark

**Affiliations:** ^1^ Matrix Biology Program, Benaroya Research Institute at Virginia Mason, Seattle, WA, United States; ^2^ Department of Clinical Sciences, Lund University Clinical Research Center (CRC), Skåne University Hospital, Malmö, Sweden; ^3^ Department of Medicine, University of Massachusetts, Worcester, MA, United States; ^4^ Department of Microbiology & Immunology, Drexel University College of Medicine, Philadelphia, PA, United States

**Keywords:** autoimmune diabetes, BB rat, hyaluronan, insulitis, pancreatic islets, T-cell receptor

## Abstract

The depleting Vβ13a T cell receptor monoclonal antibody (mAb) 17D5 prevents both induced and spontaneous autoimmune diabetes in BB rats. Here it was tested in congenic DR*Lyp/Lyp* rats, all of which spontaneously developed diabetes. Starting at 40 days of age, rats were injected once weekly with either saline, His42 Vβ16 mAb, or 17D5 mAb and monitored for hyperglycemia. Diabetes occurred in 100% (n = 5/5) of saline-treated rats (median age, 66 days; range 55–73), and in 100% (n = 6/6) of His42-treated rats (median age, 69 days; range 59–69). Diabetes occurred in fewer (n = 8/11, 73%) 17D5-treated rats at a later age (median 76 days, range 60–92). Three (27%) of the 17D5-treated rats were killed at 101–103 days of age without diabetes (17D5 no-diabetes rats). Survival analysis demonstrated that 17D5 mAb delayed diabetes onset. Saline- and His42-treated rats had severely distorted islets with substantial loss of insulin-positive cells. These rats exhibited prominent hyaluronan (HA) staining, with the intra-islet HA+ accumulations measuring 5,000 ± 2,400 µm^2^ and occupying 36 ± 12% of islet area, and severe (grade 4) insulitis with abundant infiltration by CD68+, CD3+, and CD8+ cells. The 17D5 mAb-treated rats with delayed diabetes onset exhibited less severe insulitis (predominantly grade 3). In contrast, the 17D5 no-diabetes rats had mostly normal islets, with insulin+ cells representing 76 ± 3% of islet cells. In these rats, the islet HA deposits were significantly smaller than in the diabetic rats; the intra-islet HA+ areas were 1,200 ± 300 µm^2^ and accounted for 8 ± 1% of islet area. Also, islet-associated CD68+ and CD3+ cells occurred less frequently (on average in 60 and 3% of the islets, respectively) than in the diabetes rats (present in >95% of the islets). No CD8+ cells were detected in islets in all 17D5 no-diabetes rats. We conclude that mAb 17D5 delayed diabetes in DR*Lyp/Lyp* rats and markedly reduced expression of HA and concomitant infiltration of CD68+, CD3+, and CD8+ cells. Our findings underscore the importance of refining immune suppression in prevention or intervention clinical trials to use mAb reagents that are directed against specific T cell receptors.

## Introduction

The spontaneously diabetes BB rat was described in 1978 (1) and since 1980 (2) subjected to targeted breeding to dissect the genetic mechanisms underlying islet beta cell destruction [reviewed in (3–5)]. The BB rat was found to be lymphopenic (6) which was shown by marker-assisted cross-intercross breeding, to segregate with diabetes as a single locus (2). The lymphopenia gene (7, 8) has a frame-shift mutation in the *Gimap5* gene, resulting in absence of expression of the Gimap5 anti-apoptopic protein (9, 10). Successive crosses of the lymphopenic, diabetes BB-DP rat with the diabetes resistant, non-lymphopenic BB-DR rat allowed the development of the congenic DR*Lyp/Lyp* rat. In this rat, 1) spontaneous diabetes rarely occurs before 50 days of age; 2) 100% of the rats of both sexes develop diabetes before 80 days of age; and 3) a diabetes genetic susceptibility locus, *Iddm14*, was identified proximal to the *Gimap5* mutation (11). This factor was later shown to be a TCR beta chain gene, *Tcrb-V13*, specifically the *Tcrb-V13S1A1* allele. This allele encodes the Vβ13a TCR beta chain (11–13).

DR*Lyp/Lyp* rats (BB Malmö or BBM) were used first to establish that the 17D5 TCR Vβ13a mAb (14, 15) would affect development of diabetes similar to BB-DP and BB-DR rat strains from the Worcester, MA colony (15). Second, we wanted to use this monoclonal antibody to test if islet hyaluronan (HA) is a major contributor to insulitis progression. Islet-infiltrating leukocytes are therapeutic targets in type 1 diabetes since these cells and their secreted products are thought to cause beta-cell destruction. We recently found that islet leukocytic infiltration is associated with remodeling of islet extracellular matrix (ECM), specifically accumulation of hyaluronan (HA), a major islet ECM component (16). HA, a high molecular weight polysaccharide, accumulates in inflamed tissues and is a key regulator of several aspects of inflammation including leukocyte migration and the generation of inflammatory cytokines at sites of injury (17). We showed that HA accumulation in islets precedes insulitis in individuals at high risk of type 1 diabetes as well as in diabetes-prone BB rats in the pre-clinical phase of the disease, and that immune cells enter islets only in the regions that contain large amounts of HA (18). We also found that accumulation of HA in islets determines the continuum of islet immune cell infiltrates from initial peri-insulitis to severe invasive insulitis and with progressive beta-cell loss (16, 18).

The aim of the present study was to administer the 17D5 TCR Vβ13 mAb to DR*Lyp/Lyp* rats in an attempt 1) to replicate the delay in onset or prevention of diabetes reported in BB DR rats treated with virus or the viral mimetic polyinosinic:polycytidylic acid (poly I:C) as well as in BB-DP (15) and LEW.1WR1 (19) rats; 2) to compare CD3-positive (CD3+) and CD8+ T cell and CD68+ monocyte infiltration to that previously reported in the BB rat (20) and human insulitis (21–24), and 3) to examine if islet mononuclear cell infiltration affects HA deposition in islets whether the islets were protected from insulitis, or not, by the 17D5 TCR Vβ13 mAb.

## Materials and Methods

### Animals

Heterozygous BB DR*Lyp/+* rats originated at the University of Washington (UW), Seattle, WA, USA (8, 11) and were transferred to the Clinical Research Center at Lund University in Malmö, Sweden in 2008. They were re-derived by Caesarian section into a pathogen-free facility fulfilling FELASA standards. Sentinel rats were tested quarterly for known pathogens and although positive for three strains of helicobacter at UW, the re-derived BB rat line in Malmö (BBM DR*Lyp/+*) was free of these and other known bacterial strains as analyzed by the National Veterinary Institute, Uppsala, Sweden. Rats used in these studies were in their 2–4^th^ generation of inbreeding at our facility. All rats were housed at 21–23°C with *ad libitum* access to food and water. Congenic BMM DR*Lyp/Lyp* rats were maintained in heterozygous sister-brother breeding. The project was approved by the Animal Ethical Committee in Lund University, Sweden.

### Genotyping

A panel of seven SNPs was used to genotype the rats in earmarking biopsies obtained from 21-day-old pups. Each SNP marks the presence of either BB-DP or BB-DR rat DNA. SNPs above and below the flanking markers represent BB-DR rat DNA. The BB-DP rat genetic contribution is estimated to represent about 1.4 Mbp of DP-derived DNA in the BBM DR*Lyp/+* congenic strain.

DNA was isolated by digestion for 90 min at 55°C at an Eppendorf Thermomixer (Eppendorf, Hamburg, Germany) in 10 µl/sample of 20 mg/ml Proteinase K (recombinant, PCR grade, Thermo Scientific, Waltham, MA, USA), followed by precipitation in ice cold isopropanol. After drying, the DNA pellet was dissolved in TE buffer (AppliChem, Darmstadt, Germany) and shaken overnight at 37°C. DNA (2 µl) in 384 well plates were subjected to PCR analysis with the forward and reverse primers shown in **Table 1**. Primers and TaqMan^®^ Genotyping Master Mix (Applied Biosystems, Foster City, CA, USA) in a final 3–5 µl volume were added; the 384 well plates were centrifuged before sealed and then incubated (ViiA 7 RT-PCR System, Applied Biosystems, Waltham, MA, USA).

**Table 1:** SNP genotyping of the *lyp* region in the DR*Lyp/Lyp* rats.

**Position**	**Dist. Mutation**	**Forward Primer Seq.**	**Reverse Primer Seq.**	**Rep 1 Seq. (DR+/+)**	**Rep 2 Seq. (DR*Lyp/Lyp*)**
77 630 116	-753 877	GCAGGAGAGGCTGCTATGTC	TCCACAGCATGCCAAGCT	CCACAGGAGAGTTGTG	CACAGGAGACTTGTG
78 170 204	-213 789	CCTCCCTTGGTATGTTGTGTATGAT	TGCCAGGGATAGCAGAGTGA	TCCCTTCTGTGTCCAGTAC	CCTTCTGCGTCCAGTAC
78 377 812	-6 181	GGTAAATACTTCACCCTGCTTCCA	GCTGGTTACCCCTTCTTTACTATTTGA	CATTATGGAGTTTCTAGCCTAT	ATGGAGTTTCTCGCCTAT
78 383 993	0	ACATGGGAGGGAAGGAGCTT	CATGTCTTGGTTCTGGATCTTTGAC	ACGCCCCCCATCTT	CACGCCCCCATCTT
78 386 587	2 594	GATACGGGAAAGTACAGGGTAGACT	TCAGTTTTTGAAAATGACATCTAAAGTTTCTGT	ATTCTCTCCGACCCCAGTT	ATTCTCTCCGAACCCAGTT
78 576 508	192 515	CCCTACTACCTGCCCTTTAGAAG	GACCTGCTTATTTCTGGAGTGGATT	TCTTCAGATGTACTTTTAG	CTTCAGATGTCCTTTTAG
79 071 296	687 303	CCATACCATCTCTATCATCCTGTTTCC	GGGCTACAAAACAAAGCAGAAAGTT	AATCTGGGAAAGGCA	TGAATCTGAGAAAGGCA

Distance mutation is the number of base pairs from the Gimap5 frame shift mutation (0). The forward and reverse primer sequences used to identify the SNP between DR.+/+ (wild type) and DRLyp/Lyp are shown.

### T Cell Receptor Monoclonal Antibodies

The T cell monoclonal antibodies 17D5 (14, 25) and His42 (26) directed against TCR Vβ13^a^ and Vβ16, respectively, were prepared in the Mordes’ laboratory, University of Massachusetts, Worcester, MA, USA. Two batches of 17D5 and one batch of His42 were used. The hybridoma producing 17D5 mouse anti-rat Vβ13 mAb (IgG2a) recognizes the product of the *Tcrb-V13S1A1* (Vβ13^a^) allele of the *Tcrb-V13* (Vβ13) gene (14). The hybridoma producing His42 mouse anti-rat Vβ16 (IgG2b) mAb (26) was originally the gift of Dr. Thomas Hünig. Both antibodies were prepared as ascites and purified by affinity chromatography.

### Treatment Protocol

The BBM DR*Lyp/Lyp* rats were weighed daily in the morning (07:30–08:30). Blood glucose was measured daily beginning at 40 days of age. Starting at 40 days of age rats were injected intraperitoneally (i.p.) once a week with either 0.1 mg 17D5 (n = 11) or His42 (n = 6) mAb in 0.5 ml saline, or with 0.5 ml saline alone (n = 5). Initially, six, three, and three, rats received either 17D5, His42, or saline, respectively. The rest of the rats, generated from additional breeding, received the treatment 2 months later, and were followed similarly to the first group. Treatment continued until the rats developed diabetes (defined as a glucose concentration >11.1 mmol/L). Six rats that did not receive any treatments were killed at 40 days of age. It was predetermined that DR*Lyp/Lyp* rats that remained diabetes-free at 95 days of age would be killed at about 100 days of age.

### Immunohistochemistry

Formalin-fixed paraffin-embedded pancreas tissues were serially sectioned. Consecutive sections were stained for H-E, insulin and glucagon, HA, CD68, CD3, and CD8 in different combinations. Four to six sets of serial sections were prepared per pancreas. All islets present in a stained section, on average 71 islets (range 52–99 islets), were counted per rat. The following primary antibodies were used: insulin (Dako, Glostrup, Denmark), glucagon (Sigma-Aldrich, St Louis, MI, USA), and CD68 (clone ED1, Bio-Rad, Hercules, CA, USA), at dilutions 1:500, 1:2,000, and 1:100, respectively. Antibodies to CD3 (clone SP7, Abcam, Cambridge, UK) and CD8 (Abcam) were used at dilution 1:150. Staining for HA was performed as described (16, 18). Sections were incubated overnight with the primary antibodies and then for 1 h with the following secondary antibodies at dilution 1:400: Alexa Fluor^®^ 488 conjugated goat anti-rabbit IgG (Invitrogen), Alexa Fluor^®^ 568 conjugated goat anti-mouse IgG (Invitrogen), and Cy™2 donkey anti-guinea pig IgG (Jackson Immunoresearch). The stained sections were mounted with VECTASHIELD Vibrance with DAPI (a nuclear counterstain) Antifade Mounting Medium (Vector Laboratories, Burlingame, CA, USA).

### Quantification of Insulin- and HA-Stained Areas

Islet insulin-stained areas were measured using ImageJ supported by the National Institutes of Health (https://imagej.nih.gov/ij/index.html). The measurement of total and intra-islet HA-positive (HA+) areas was performed using whole-section imaging as described (16, 18).

Whole-section imaging was performed using a NanoZoomer Digital Pathology slide scanner (Hamamatsu; Bridgewater, NJ, USA). Slides were scanned in bright field with a 20× objective and the digital images imported for analysis using HALO image analysis software (Indica Labs, Albuquerque, NM, USA). Islets were identified by their staining for synaptophysin (SYN). All the islets present in the rat pancreas sections were analyzed. The intra-islet HA+ area represents the HA located within islet area bordered by the endocrine side of the peri-islet capillaries. The total islet HA area is the sum of the intra-islet HA+ and the HA+ area measured around the islets at a 5-nm distance from the endocrine side of the peri-islet capillaries (16). The outer border of this area was delineated using the HALO annotation tool. The stained areas were measured using HALO software. The percent islet HA+ area is calculated as intra-islet HA+ area/islet area × 100.

### Evaluation of Insulitis

Insulitis was quantified as described (16, 18, 27). The sections were independently scored by two investigators unaware of the treatment status of the animals as follows: Grade 0, no infiltration; Grade 1, leukocytes present around islets (peri-insulitis) or ≤15 dispersed leukocytes within islets; Grade 2, leukocytes infiltrating <25% of the islet area; Grade 3, leukocytes occupying 25–50% of the islet area; Grade 4, leukocytes diffusely present throughout the islet and end-stage islets devoid of beta cells. The distribution of macrophages and cytotoxic T cells was evaluated in sections stained for the immune markers CD68, CD3, and CD8. All the islets and immune cells stained for these markers present in the sections were counted, and the prevalence of islets with CD68+, CD3+, or CD8+ cells and the number of these cells per islet were determined.

### Statistical Analysis

Parametric data are given as arithmetic means ± SD. Diabetes-free survival is presented as the median and groups were compared using Kaplan-Meier methodology; equality of survival distributions was tested by log rank statistic (GraphPad^®^). The significance of the difference between two or more groups of data was evaluated using the Mann–Whitney U test or ANOVA. A *p* value of less than 0.05 was considered statistically significant. All analyses were performed using GraphPad Prism version 8.00 for Windows (GraphPad Software, San Diego, CA, USA).

## Results

### Treatment With 17D5 mAb Delayed or Prevented Diabetes Onset in BBM DR*Lyp/Lyp* Rats

**Figure 1:**
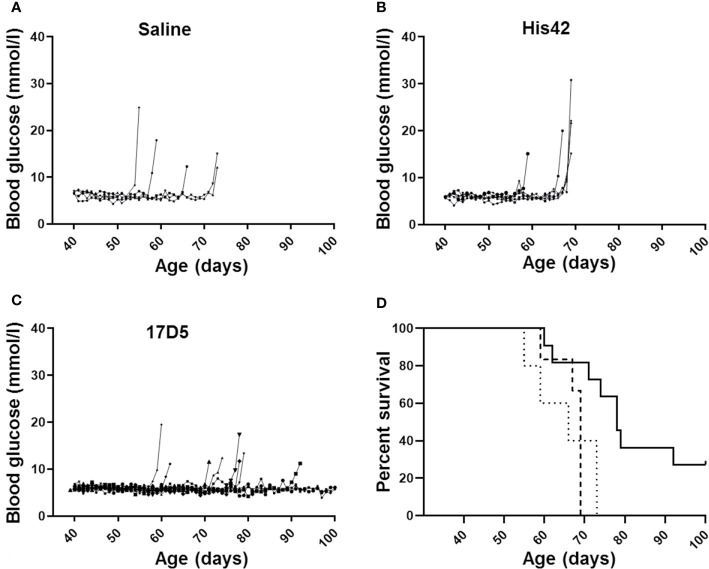
Daily blood glucose levels measured daily and age at diagnosis of diabetes in DR*Lyp/Lyp* rats receiving either saline (**A**, n = 5), monoclonal antibody His42 (**B**, n = 6), or monoclonal antibody 17D5 (**C**, n = 11). The saline treated DR*Lyp/Lyp* rats developed diabetes between 55 and 73 days of age (median 66 days). The His42-treated rats developed hyperglycemia between 59 and 69 days of age (median 69 days). In contrast, diabetes occurred in fewer rats treated with 17D5 (n = 8/11), between 74 and 92 days of age (median 76 days). Kaplan Meier analysis in **(D)** showed that survival times in the saline (dotted line) and His42 (dashed line) groups were statistically similar whereas survival in the 17D5 group (solid line) was prolonged (*P* < 0.001).

All saline treated DR*Lyp/Lyp* rats (n = 5/5) developed diabetes between 55 and 73 days of age (median 66 days; **Figure 1A**). The average blood glucose concentration at diagnosis was 16.4 ± 5.3 mmol/L. Administration of the His42 mAb did not affect diabetes onset; all His42-treated rats (n = 6/6) developed hyperglycemia between 59 and 69 days of age (median 69 days, **Figure 1B**). The average blood glucose level at diagnosis was 20.8 ± 5.8 mmol/L. In contrast, diabetes occurred in fewer rats treated with 17D5 (n = 8/11, 73%), and the age at onset was delayed (median 76 days, range 74 to 92 days, *P* < 0.05, **Figures 1C, D**). The blood glucose concentration at diagnosis in the eight 17D5-treated rats developing diabetes (17D5 delayed group) was 13.5 ± 3.2 mmol/L, which was slightly (~3 mmol/L) lower than in the combined cohort of saline- and His42-treated rats (*P* < 0.05). Five of the eight 17D5 rats did not develop hyperglycemia until 74, 78 (n = 2), 79, and 92 days of age, respectively. Remarkably, three 17D5-treated rats maintained normal blood glucose until 101 (n = 1) and 103 (n = 2) days of age, at which time the rats were killed per protocol (17D5 no-diabetes group). The blood glucose levels at the day of killing were 5.6, 6.6, and 5.3 mmol/L, respectively. Survival analysis of the entire cohort showed that the 17D5-treated rats had a longer diabetes-free survival (*P* < 0.001) compared to the other groups (*P* > 0.05).

### Growth in Saline-, His42-, and 17D5-Treated DR*Lyp/Lyp* Rats

The body weight (g) curves indicated that growth was not affected by either mAb treatment. At disease onset, the 17D5-treated female and male rats weighed 201 ± 21 g and 309 ± 18 g, respectively, which were comparable to those of the female and male rats in the combined saline and His42 groups (188 ± 9 and 292 ± 10 g). Similarly, the average growth rate (g/day) in the female and male rats did not differ significantly among the 17D5-treated (n = 11) and the combined saline and His42 groups (n = 11, data not shown).

### Preservation of Islet Architecture and Beta Cells in 17D5 No-Diabetes DR*Lyp/Lyp* Rats

**Figure 2:**
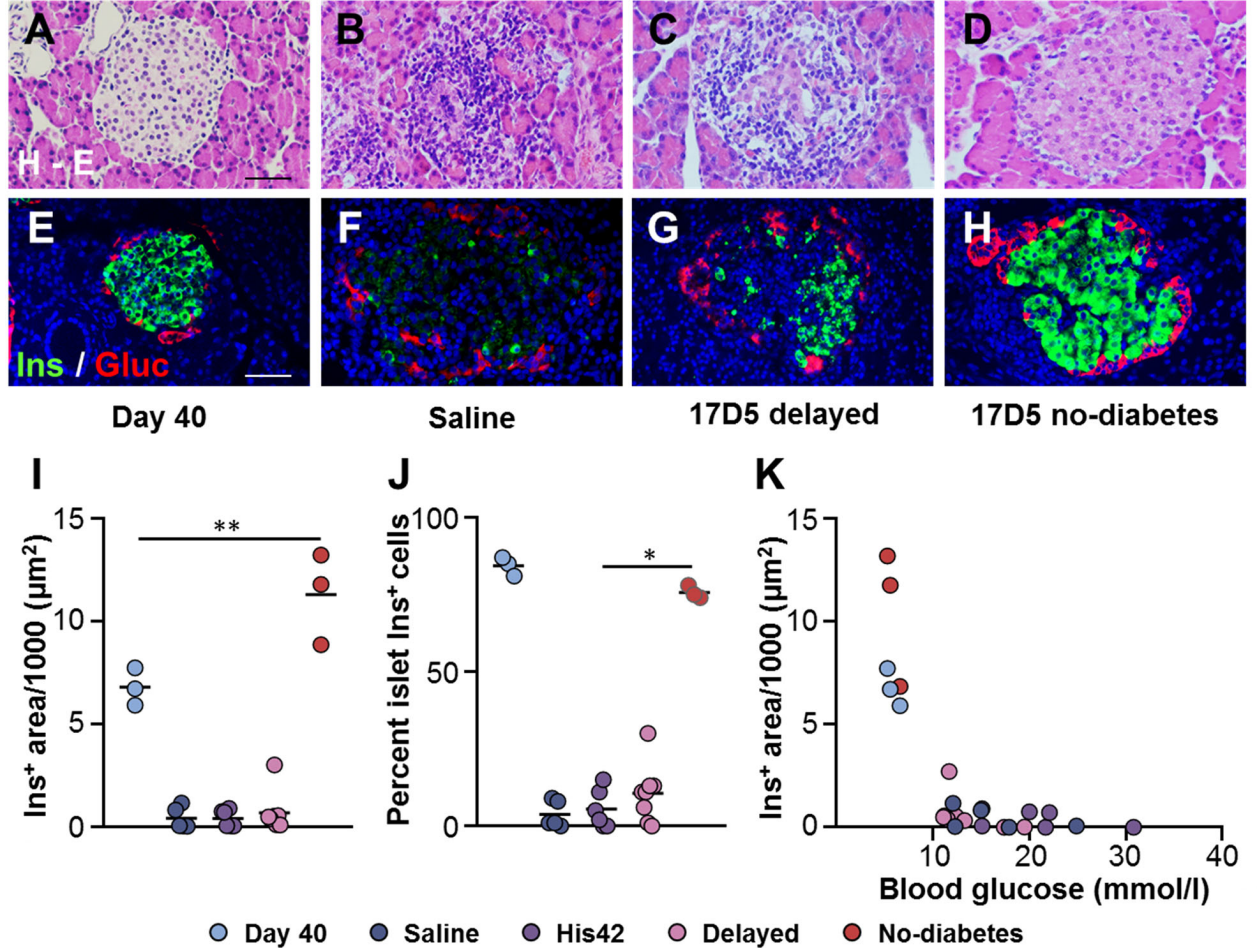
Preservation of islet morphology in the 17D5-no diabetes DR*Lyp/Lyp* rats. **(A–D)** Hematoxylin-Eosin (H-E) staining in pancreas tissues from rats in the indicated groups. **(E–H)** Immunohistochemistry for insulin (Ins, green) and glucagon (Gluc, red). Scale bars, 50 µm. To avoid redundancy, images from His42-treated rats are not shown. **(I)** Morphometric quantification of islet Ins+ areas. **(J)** Relative proportion of islet Ins+ cells. **(K)** Ins+ areas plotted as a function of blood glucose levels. Each circle denotes an individual rat; dark slate blue, saline (n = 5); purple, His42 (n = 6); pink, 17D5 delayed (n = 8); red, 17D5 no-diabetes (n = 3); light slate blue, 40 day old (n = 3). Data are mean values of measurements obtained for each rat. The solid horizontal lines in **(I, J)** indicate the mean of the measurements in each group. *P < 0.05, 17D5 no-diabetes *vs* saline, His42, or 17D5 delayed; **P < 0.01, 17D5 no-diabetes *vs* saline, His42, 17D5 delayed, or day 40; Mann-Whitney U test.

At 40 days of age, DR*Lyp/Lyp* rat islets typically exhibit the endocrine cell composition and organization observed in normoglycemic rodents, with the insulin+ cells being the majority of the endocrine cells (83 ± 4%) located in the center, surrounded by a halo of glucagon+ cells (**Figures 2A, E**). At the time of clinical onset, the diabetes rats in the saline, His42, and delayed diabetes groups exhibited substantial loss of beta cells (**Figures 2B, C, F, G, I**) with the remaining insulin+ cells accounting for 7 ± 8% of the islet cells. The insulin-stained areas measured 500 ± 640 µm^2^ and occupied 0.03 ± 0.02% of the pancreas area. In 18 of 19 diabetes rats, the proportion of insulin+ cells varied from 0 to 15%. Interestingly, in one rat in the delayed diabetes group, which developed hyperglycemia at 78 days of age, one third of the islets still contained insulin+ cells accounting, on average, for 32% of islet cells. In addition, in this rat, the glucagon+ cells had maintained their peripheral location in islets (**Figure 2G**), indicative of some degree of preserved islet architecture. In contrast to diabetes rats, islet architecture and cell composition were maintained in the 17D5 no-diabetes rats (**Figures 2D, H**). The islet insulin+ areas in these rats were 30- and 21-fold larger than in the control (saline- and His42-treated) and 17D5 delayed diabetes rats, respectively, and the insulin+ cells represented 76 ± 3% of all islet cells (**Figures 2I–K**), which is comparable to that in the normoglycemic DR*Lyp/Lyp* rats at 40 days of age (*P* > 0.05).

### Prevention of Invasive Insulitis in 17D5 No-Diabetes DR*Lyp/Lyp* Rats

The rats in the saline- and His42-treated groups exhibited severe insulitis (**Table 2**). In these rats, 94% of the islets (range 88–100%) were penetrated by dense inflammatory cell infiltrates. The rats in the 17D5 delayed group overall exhibited a lower degree of immune cell infiltration as compared to the two other diabetes groups. In the former, on average, the prevalence of islets with severe insulitis (grade 4) was 68 ± 10%, which was lower than that in the latter (94 ± 5%, *P* < 0.005). Also, in the 17D5 delayed rats, on average 18% of the islets exhibited no or limited islet infiltration, compared to only 3% of the islets in the control diabetes rats. In contrast, in the three 17D5-treated normoglycemic rats, on average, 83 ± 3% of the islets did not show any evidence of insulitis. Peri-insulitis or scarce immune cells in the islet periphery were observed in 11 ± 2% of the islets, while only 6% of the islets exhibited infiltrating immune cells that had spread throughout the islet.

**Table 2:** Evaluation of insulitis in the DR*Lyp/Lyp* rats.

Group	Rat (n)	% Islets with insulitis				
		Grade 0	Grade 1	Grade 2	Grade 3	Grade 4
**Saline**	5	1	1	1	2	95
**His42**	6	0	0	3	4	93
**17D5 delayed**	8	2	5	8	13	71
**17D5 no-diabetes**	3	83	11	4	1	0

**Figure 3:**
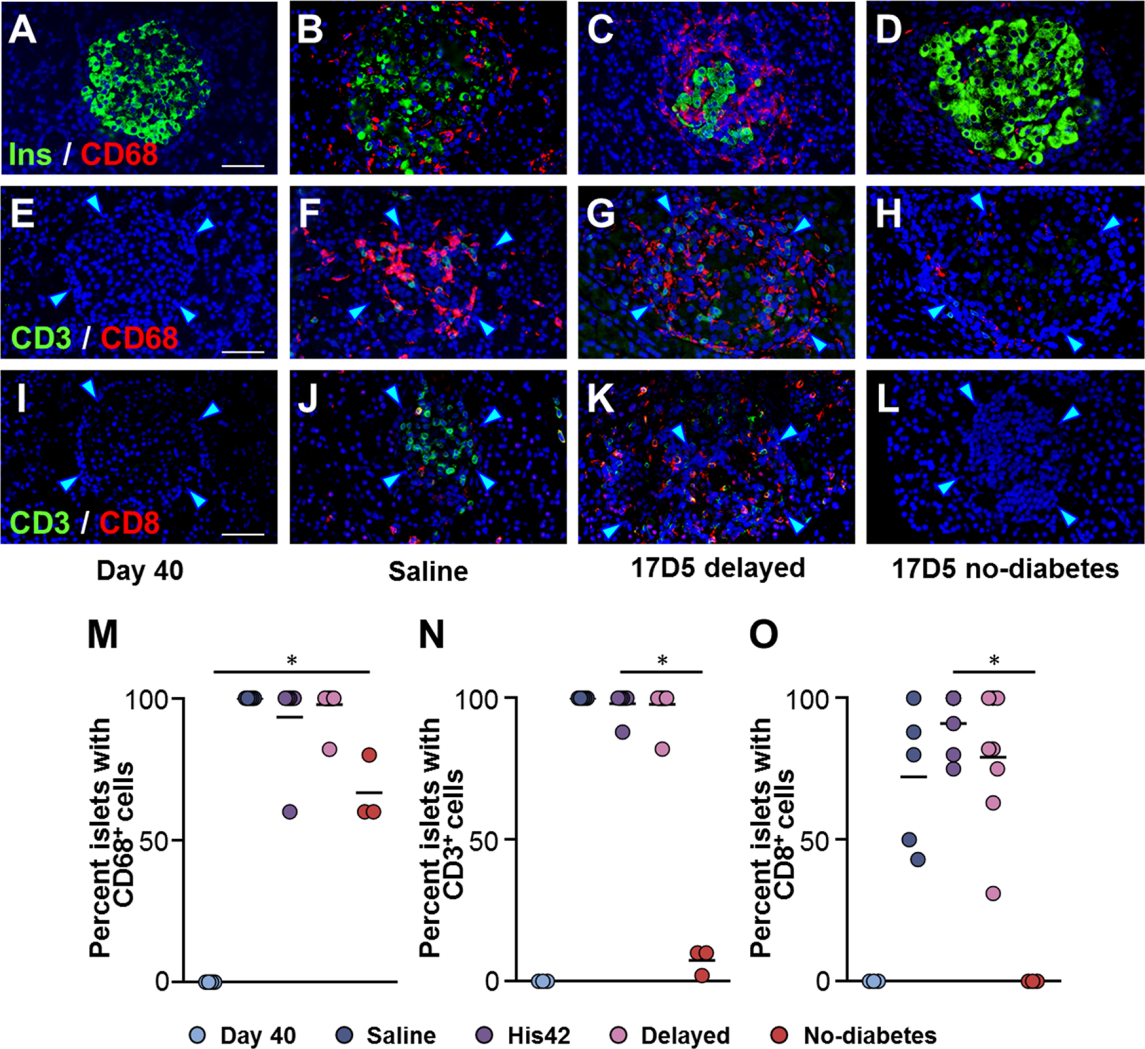
Occurrence of insulitic macrophages and lymphocytic cells in DR*Lyp/Lyp* rats. **(A–D)** Immunohistochemistry for insulin (Ins, green) and the macrophage marker CD68 (red) in the indicated groups. Immunostaining for the T cell marker CD3 (green) and either CD68 **(E–H)**, or the cytotoxic T cell marker CD8 **(I–L)**. Scale bars, 50 µm. To avoid redundancy, images from His42-treated rats are not shown. The arrowheads point to the islet border. Prevalence of islets with insulitic CD68 **(M)**, CD3 **(N)**, and CD8 **(O)** cells. Each circle denotes an individual rat; dark slate blue, saline (n = 5); purple, His42 (n = 6); pink, 17D5 delayed (n = 8); red, 17D5 no-diabetes (n = 3); light slate blue, 40 day old (n = 3). Data are mean values of measurements obtained for each rat. The solid horizontal lines indicate the mean of the measurements in each group. *P < 0.05, 17D5 no-diabetes *vs* saline, His42, 17D5 delayed, or day 40; Mann-Whitney U test.

Insulitic CD68+ cells were absent in the DR*Lyp/lyp* rats at 40 days of age (**Figure 3A**), while they were abundant in the diabetic rats (25 ± 16 cells/islet), where they occurred in the vast majority of the islets (**Figures 3B, C, M**). Islet-associated CD68+ cells were observed in the pancreata of the three 17D5 no-diabetes rats (**Figure 3D**). However, these cells were present in significantly fewer numbers than in the diabetic rats (1–7 cells/islet, *P* < 0.05).

Since exposure to 17D5 mAb was expected to deplete T cells, we examined the presence of CD3+ and CD8+ T cells in islets of 17D5-treated rats (**Figures 3E–L**). CD3+ cells were observed in >95% (range 88 to 100%) of the islets in all diabetes rats with a frequency that did not differ between the 17D5 delayed and saline- and His42-treated rats (18 ± 3 *vs* 20 ± 6 CD3+ cells/islet, respectively, *P* > 0.05). However, only 3% of the islets in the 17D5 no-diabetes rats exhibited CD3+ cells (**Figure 3N**) that occurred in significantly fewer numbers than in the other three groups (0.5 ± 0.6 cells/islet, *P* < 0.05). CD8+ cells were present in all the diabetes rats and were observed in 81% (range 43 to 100%) of their islets. In contrast, no CD8+ cells were detected in the vicinity or within islets in the three 17D5-treated normoglycemic rats (**Figure 3O**). Thus, the 17D5 mAb has successfully prevented immune cell infiltration and beta cell destruction in these rats.

### Treatment With 17D5 Prevents the Formation of Large HA Deposits in Islets in 17D5 No-Diabetes DR*Lyp/Lyp* Rats

**Figure 4:**
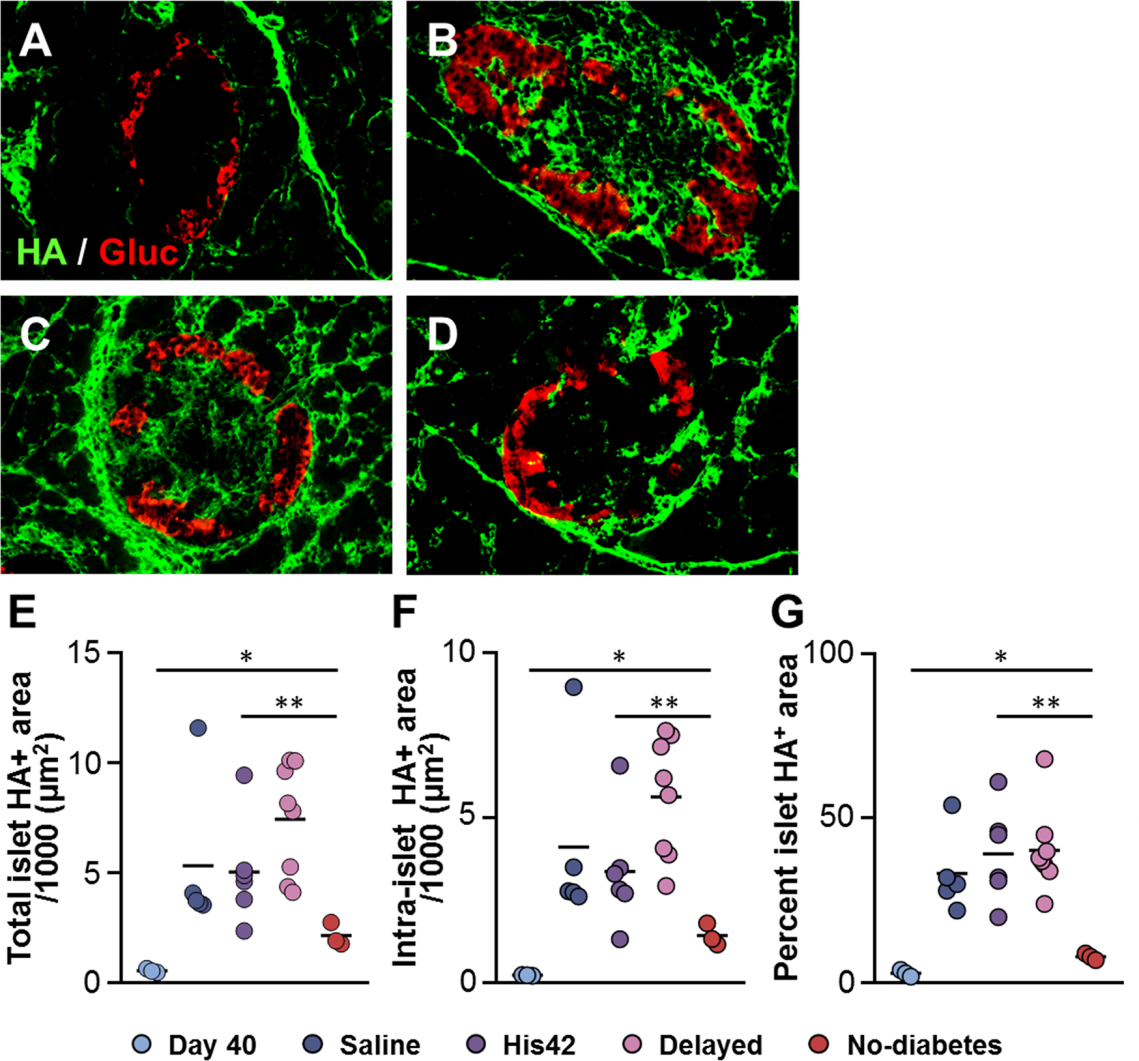
Reduction in islet hyaluronan (HA) in 17D5 no-diabetes DRLyp/Lyp rats. **(A–D)** HA (green) and glucagon (Gluc, red) staining in islets from DRLyp/Lyp rats at 40 days of age **(A)**, and from rats in the saline **(B)**, 17D5 delayed **(C)**, and 17D5 no-diabetes **(D)** groups. Scale bar, 50 µm. To avoid redundancy, images from His42-treated rats are not shown. Morphometric quantification of total **(E)** and intra- **(F)** islet HA+ areas. **(G)** Relative proportion of islet areas stained for HA. Each circle denotes an individual rat; dark slate blue, saline (n = 5); purple, His42 (n = 6); pink, 17D5 delayed (n = 8); red, 17D5 no-diabetes (n = 3); light slate blue, 40 day old (n = 3). Data are mean values measurements obtained for each rat. The solid horizontal lines indicate the mean of the measurements in each group. *P < 0.05, 17D5 no-diabetes *vs* day 40; **P < 0.01, 17D5 no-diabetes *vs* saline, His42, or 17D5 delayed; Mann-Whitney U test.

In line with our previous findings (18), HA staining was scanty in islets of DR*Lyp/Lyp* rats at 40 days of age (**Figure 4A**). The older rats that developed diabetes exhibited substantial accumulation of HA in their islets, both within and at islet periphery (**Figures 4B, C**), which is consistent with our previous observation of the formation of large HA deposits in DR*Lyp/Lyp* rats exhibiting hyperglycemia (18). In these rats, the intra-islet HA+ accumulations measured 5,000 ± 2,400 µm^2^ (**Figure 4F**), occupying on average 36 ± 12% of the islet area (**Figure 4G**). In contrast, the islet HA deposits were significantly smaller than in the 17D5-treated DR*Lyp/Lyp* rats that remained normoglycemic at the end of the period of observation. In these rats, the intra-islet HA+ areas were 1,200 ± 300 µm^2^ and accounted for 8 ± 1% of the islet area (**Figures 4D–G**). However, in the 17D5-no diabetes group, the HA-stained areas were 5-fold larger than in their normoglycemic littermates at 40 days of age, while the islet size increased only 70%.

## Discussion

The major finding of this study is that the Vβ13 T cell receptor mAb 17D5 delayed the onset of diabetes in congenic DR*Lyp/Lyp* rats. It confirms previous observations in spontaneously diabetes BBDP rats (15) and LEW.1WR1 rats in which diabetes was induced by poly I:C or viral infection (13). While all DR*Lyp/Lyp* rats injected with saline developed diabetes between 55 and 73 days of age, remarkably, the 17D5 monoclonal mAb not only delayed the onset but also prevented diabetes up until 100 days of age in a sizable and significant proportion of the treated animals. Interestingly, 17D5-treated rats had lower blood glucose at diagnosis than those in the saline- or His42-treated groups. Diabetes still developed rapidly in some 17D5-treated rats, but the severity of hyperglycemia at onset was reduced, perhaps due to fewer beta cell-specific cytotoxic T cells. The 17D5 TCR Vβ13a mAb should therefore prove useful to dissect the mechanisms of beta-cell killing.

The three treated rats that remained diabetes-free by 100 days of age had normal blood glucose associated with a reduction in CD3+ and CD8+T cells as well as in HA staining. Some infiltration of mononuclear cells was observed in them so it cannot be excluded that, if these rats had been allowed to survive, they might have developed infiltration of CD3+ and CD8+ T cells along with CD68+ cells and expanded their islet HA deposits. The data suggest that treatment with 17D5 likely needs to be continuous as opposed to a brief treatment to prevent diabetes. It is unlikely that the 17D5 mAb treatment was curative, and further studies are warranted to explore the mechanisms by which it prevents both islet mononuclear cell infiltration and progressive accumulation of HA. It would also be important to titrate the 17D5 mAb to determine a suitable and informative dose level for future experiments.

Differences between the present study and earlier studies of the 17D5 mAb are noted. In spontaneously diabetes BBDP/Wor rats from the Worcester colony, the same mAb using the same protocol (weekly treatment from 45 to 100 days of age) completely prevented diabetes (15). However, diabetes in the BBDP/Wor rat is more indolent in onset and not 100% penetrant; not all control animals in the 17D5 treatment study developed diabetes. In studies of autoimmune diabetes that is induced in LEW1WR1 rats by viral infection or poly I:C, treatment was instituted before the induction of autoimmunity (at ~21 days of age) and given three times weekly (15). In that study most animals were protected. Hence, in regard to partial protection afforded by 17D5 in the DR*Lyp/Lyp* rat, it remains to be determined if the treatment was sufficiently intense (in terms of dose, frequency, or both), or if it was started too late. The present study protocol required killing any rat that survived 100 days of age; at that time point the blood glucose was within the normal range, and the HA deposition and insulitis scores were also markedly reduced. The variable reduction in peri-insular and islet infiltrating CD68+ cells by the 17D5 mAb suggest that the mAb was not fully effective at preventing autoimmune inflammation. Taken together, the data suggest that treatment with 17D5 mAb might have reduced the migration of inflammatory cells into islets and reduced beta cell death.

We have speculated that the migration and accumulation of CD3+ and CD8+ cells may be related to the presence of HA. This hypothesis is based on our recent work showing that, in both humans and BB rats in the preclinical phase of the disease, leukocytes that infiltrate islets do so exclusively in the regions that are rich in HA, and that progressive accumulation of HA in islets is associated with advancement to severe insulitis (18). Although the 17D5 Ab did not deplete the population of islet infiltrating CD8+ cells in the delayed-diabetes rats, it can be speculated that 17D5 might modify or inhibit their interactions with HA or other local cells, and thus reducing their capacity for migration and hence the severity of insulitis in these rats. Our recent studies indicated that in BB rats in the pre-clinical phase of the disease, the extent of the HA deposition is associated with the level of the insulitic cell infiltration, in particular that of grades 1–3. The present study reinforces this finding; the 17D5 no-diabetes rats did not develop the extensive HA deposits observed in the diabetes rats. That the size of HA deposits in the 17D5 delayed-diabetes rats was comparable to that in the saline and His42 diabetes rats, in spite of a lower degree of insulitis (grade 3 *vs* grade 4) is also in line with our previous observation that the size of the islet HA accumulations does not increase further with the progression from grade 3 to grade 4 insulitis.

The reduction in islet HA along with the attenuation of insulitis in the three normoglycemic 17D5-treated BB rats further indicates a relationship between the presence of VB13a+ T cells, invasive insulitis and the accumulation of HA.

In spite of limiting HA accumulation, the effective 17D5 mAb treatment did not fully prevent the deposition of HA in islets; the no-diabetes DR*Lyp*/*Lyp* rats still exhibited HA+ areas that were 5-fold larger than those measured in the non-diabetic 40-day-old DR*Lyp/Lyp* rats, while the beta cell areas were expanded only by 70%. We recently reported that in the diabetes prone DR*Lyp/Lyp* rats, the initial induction of HA synthesis and deposition in islets takes place prior to insulitis (18). HA synthesis may therefore not be dependent on the presence of infiltrating cells but could rather be due to other factors generated locally or reaching the islets *via* circulation (18). We also showed that the islet HA deposits expanded with time, which was associated with the progression to invasive insulitis. The fact that the treatment with a mAb expected to deplete or inhibit T cells expressing one specific particular TCR beta chain suggests that continual HA accumulation in islets could be related to the presence of insulitic T cells. It is conceivable that sufficient depletion of pathogenic T cells targeting beta cells can, directly or indirectly, limit the progressive deposition of pro-inflammatory HA in islets. Absence or reduction in the number of islet-infiltrating T cells would result in a lower concentration of locally released pro-inflammatory cytokines, most of which are reported to induce HA synthesis (28). In addition, previous work has indicated that, at least *in vitro*, activated T cells and monocytes upregulate the expression of HA synthesizing enzymes (29). Therefore, it could be that fewer infiltrating T cells would reduce the pool of HA-producing cells in islets and subsequently the amount of islet HA. The formation of large HA deposits in the diabetes rats in the present study is in line with our previous observations (18). Whether the islet HA-rich ECM present in the 17D5-treated no-diabetes rats has proinflammatory properties remains to be determined.

A strength of the present preclinical prevention study is the robust and reliable onset of spontaneous autoimmune diabetes that is fully penetrant, at a limited time window between 55 and 73 days of age in the control DR*Lyp/Lyp* rats. Importantly, the line of the DR*Lyp/Lyp* rats used in this study is not responsive to treatments directed at preventing diabetes (27, 30–34). Also, the abrupt development of hyperglycemia over the course of 24 h provides an unambiguous end-point. The rapid progression to diabetes in the DR*Lyp/Lyp* rats is therefore useful in any attempt to delay or prevent diabetes. In addition, the depleting anti-Vbeta16 His42 mAb did not affect diabetes penetrance whereas the depleting anti-Vbeta13 17D5 mAb did.

Potential weaknesses of the study include the starting point for treatment at 40 days. Our BBM DR*Lyp/Lyp* rats subjected to optical projection tomography, morphometry and deterioration of beta cell function and mass, and intra-islet blood flow that preceded insulitis (18). It cannot be excluded that the treatment with 17D5 would have been more effective if initiated earlier than 40 days of age. Another potential weakness is the lack of a measure of invasivity of CD3+ and CD8+ T cells in relation to HA. However, this would have required killing of animals prior to the clinical onset of diabetes. For example, DR*Lyp/Lyp* rats with impaired glucose tolerance 1 day before the clinical onset would have been more informative than rats with fulminant diabetes.

The present study of spontaneously diabetes BB DR*Lyp/Lyp* rats has demonstrated that the anti-TCR Vβ13 mAb 17D5 delays the onset of diabetes in comparison with control saline- and His42-treated rats. This is the first time that we observe diabetes prevention in the congenic DR*Lyp/Lyp* rats. The effect in 30% of the rats is still important when considering individuals at high risk for autoimmune type 1 diabetes. In the recent secondary prevention trial with an anti-CD3 Ab, there were also responder and non-responder subjects observed (35). Human TCR specific mAb may have to be developed as part of a personalized medicine and used in combinatorial rather than a traditional monotherapy. Among rats without diabetes at 100 days of age there was a reduction in CD3+ and CD8+ T cells, reduction in CD68+ cells, and reduced HA deposition. DR*Lyp/Lyp* rats and type 1 diabetes patients exhibit similarities in the development of insulitis, which underscore the importance of refining immune suppression clinical trials with anti-CD3 intervention (36) or secondary prevention (35) with antibodies against specific T cell receptors.

## Data Availability Statement

The original contributions presented in the study are included in the article/supplementary material. Further inquiries can be directed to the corresponding authors.

## Ethics Statement

The animal study was reviewed and approved by the Animal Ethical Committee in Lund University, Sweden.

## Author Contributions 

The protocol was developed by ÅL, MF, and LF in consultation with JM and EB. LF, MF, and AM carried out the treatment and collected all data while AR was responsible for all genotyping to select eligible DR*Lyp/Lyp* rats. MB planned and carried out the histological analyses together with LF. ÅL drafted the manuscript and revisions were done together with MB, JM, EB, and LF. All authors contributed to the article and approved the submitted version. ÅL is the guarantor of this work.

## Funding

The study was supported by the Swedish Research Council (grants 2016-01792 to ÅL), the Diabetesfonden, the SUS Fund, by a Pilot Project to MB from the NIAID Cooperative Study Group for Autoimmune Disease Prevention Innovative Study (U01 AI101990) and in part by grants 1-16-ICTS-086 and 1-19-ICTS-076 from the American Diabetes Association (to JM and EB), AI 39095 from the National Institutes of Health (to JM), and by the Swedish Research Council (Strategic Research Area Exodiab, Dnr 2009-1039) as well as the Swedish Foundation for Strategic Research (Dnr IRC15-0067).

## Conflict of Interest

The authors declare that the research was conducted in the absence of any commercial or financial relationships that could be construed as a potential conflict of interest.
